# Postural loads during walking after an imbalance of occlusion created with unilateral cotton rolls

**DOI:** 10.1186/1756-0500-3-141

**Published:** 2010-05-25

**Authors:** Simona Tecco, Antonella Polimeni, Matteo Saccucci, Felice Festa

**Affiliations:** 1 Department of Oral Sciences, University G.D'Annunzio, Chieti/Pescara, Italy; 2 University La Sapienza, Roma, Italy; 3 Unit of Orthodontics, University La Sapienza, Roma, Italy

## Abstract

**Background:**

It was showed that stomatognathic functions correlate with alterations in locomotion, that are detectable through the analysis of loading during walking. For example, subjects with symptoms of Temporomandibular disorders (TMDs) showed a significant higher load pressure on the two feet, respect to health subjects, when cotton rolls were inserted. This previous study appeared to suggest that the alteration of postural loads associated to a particular alteration of stomatognathic condition (in this case, the cotton rolls inserted between the two dental arches) is detectable only in TMD's subjects, while it resulted not detectable in health subjects, because in that study, health subjects did not show any significant alteration of postural loads related to the different stomatognathic tested conditions. In other words, in that previous study, in the group of health subjects, no significant difference in postural loads was observed among the different test conditions; while TMD subjects showed a significant higher load pressure on the two feet when cotton rolls were inserted, respect to all the other tested conditions. Thus, the aim of this study was to better investigate these correlations in health subjects without TMD's symptoms, testing other different intra-oral conditions, and to verifywhether an experimentally induced imbalance of occlusion, obtained putting an unilateral cotton roll, could cause an alteration of postural loading on feet during walking.

**Findings:**

In a sample of thirty Caucasian adult females (mean age 28.5 ± 4.5), asymptomatic for TMDs, when a cotton roll was positioned on the left or the right sides of dental arches, so causing a lateral shift of the mandible, the percentage of loading and the loading surface of the ipsi-lateral foot, left or right, were found to be significantly lower than in habitual occlusion (p < 0.05). Males were not included because of their different postural attitude respect to females. Further studies in a sample of males will be presented.

**Conclusions:**

This study showed that in health subjects without TMD's symptoms, an experimentally induced imbalance of the occlusion, obtained through an unilateral cotton roll, is associated to detectable alterations in the distribution of loading on feet surface, during walking.

## Background

This study adds new data on a previous investigation, recently published, [1] in which it was showed, in a group of Caucasian adult females with TMD's symptoms, that the the interposition of two cotton rolls between dental arches causes an increase of the percentage of loading on feet during locomotion, that can not be observed in subjects without TMD's symptoms. However, this previous study does not clarify whether these types of correlations are proper of TMDs patients, or are detectable also in subjects without TMD's symptoms, that could have importance for a correct diagnosis of postural disorders.

Now, we are able to add new data to these previous findings, about the changes in the loading on feet, during walking, in health subjects without TMD's symptoms.

In this study, an imbalance of the occlusion was experimentally induced in agroup of health subjects without TMD's symptoms, through an unilateral cotton rollinterposed between the upper and the lower teeth, thus generating an asymmetric imbalance of the occlusion. The postural loading on feet during walking was then investigated.

This protocol was performed after the first study [1] from the same authors, in which some correlations between the locomotion and stomatognathic functions were observed in subjects affected by TMD's symptoms, respect to health subjects. The rationale of this new protocol was that these types of correlations can be detected not only in subjects with TMD's symptoms, as observed in the previous research [1], but also in health subjects without TMD's symptoms. For this, this new protocol was aimed to investigate health subjects.

The background of this present protocol is that other researchers in literature found some correlations between walking and occlusion in health subjects without TMD's symptoms.

For example, Flavel et al. (2003) [2] observed that the locomotion is always followed by rapid deceleration of the downward movement of the head and slightly less rapid deceleration of the downward movement of the mandible, while no tooth contact occurred in any forms of gait at any inclination;the movement of the mandible seemed to depend on the nature and velocity of the locomotion, probably due to the passive soft-tissue visco-elasticity [3] and the stretch reflexes [4] in the jaw-closing muscles.

Evidence (in cats) was also presented that the electromyographic activity of the jaw closing muscles increases during upwards movements of the head during walking and decreases as the head falls [5].

Fujimoto et al. [6] also suggested that the change of mandibular position can affect the gait stability, because the gait cycle and the coefficient of variation and gait velocity changed significantly in 5 mm opening position from the intercuspal position, and in 5 mm left and 5 mm right position from the 3 mm opening position at fast speed.

Finally, it was also demonstrated that a change in the postural stability of the subject, given for example, by a knee pathology, can generate changes in the function of trunk, neck and masticatory muscles, suggesting an interrelationship between masticatory function and body posture [7].

On the base of this background, the aim of this study was to verify whether, in health subjects without TMD's symptoms, an experimentally induced imbalance of occlusion, obtained putting an unilateral cotton roll, can be associated to an alteration of postural loading on feet during walking.

## Materials and methods

### The Baropodometer

**Figure 1:**
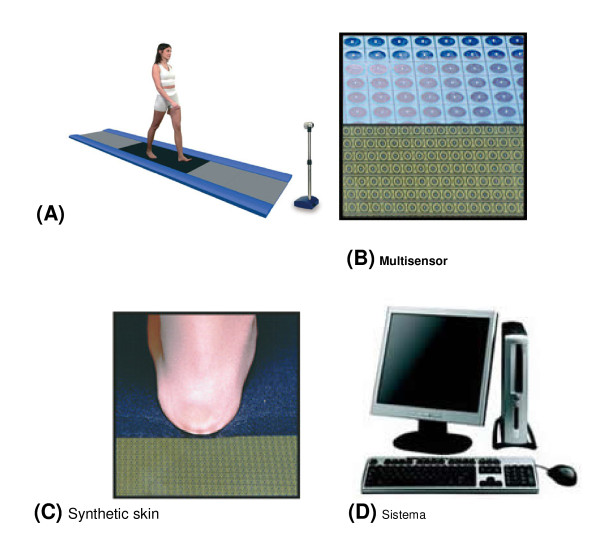
**The Baropodometer**. **(A) **Postural platform; **(B) ***SensorOne version*: the modules that employ this type of sensor assure accurate capture of surface and load expressions every square centimetre. *MultiSensor version*: the modules that employ this type of sensor assure accurate capture of surface and load expressions every square 0.25 centimetre. The *MultiSensor system *can integrate the diagnosis with the dynamic load assessment. **(C) **Synthetic skin: a special material is used to enhance the levels of sensitivity during particular pressure analyses on the surface and also around the foot. **(D) **System Intel Pentium IV/Win XP Pro 512 Mb Ram/HD 80 Gb Pressure plate 5 dpi Frequency 200 Hz max Repeatability ± 1.5% Load: 5 ÷ 1000 Kg

On 1996, it was introduced the Electronic Baropodometer (Diagnostic Support S.r.l. Via Dora 1 - 00198 Roma, Italy) (Figure 1), a patented modular platform, with the possibility to connect several modules together, obtaining a full length up to cm 240 (7,90 ft). This system allows the analysis of the walk in a natural way. The acquisitions are based detecting each cm square of the plantar surface contact. On 2006 it was introduced an advanced version of the modular technology based on an high resolution platform, realizing the modularity beyond that length also in width. This new system (Electronic Baropodometer MultiSensor) (Figure 1) uses four sensors each cm square and is available with a width of 40 cm (15,75 in) or 80 cm (31,50 in) and a length up to 320 cm (1,50 ft). While integrating optical devices and foot pressure platform, the system is named D.B.I.S. Digital Biometry Images Scanning (including hardware and software).

### The sample

Thirty Caucasian adult females were enrolled in the study after signing an informed consent form. They were selected as asymptomatic subjects for TMDs, assessed according to the American Academy of Orofacial Pain [8]. This group was partly formed by the same subjects of the control group investigated in a previous cited study from the same group [1], but in that study they were investigated in different conditions: only with two symmetric cotton rolls between dental arches. In this new study we add some findings observed when other test conditions were investigated. We add new data concerning the conditions in which the subjects are put with only one cotton roll positioned in the right or in the left sides of dental arches. The use of only one cotton roll, instead of two symmetric cotton rolls (as in the previous study), causes an asymmetric imbalance of the occlusion caused by the shift of the mandible versus one side or another when only a cotton roll is inserted between dental arches. When two symmetric cotton rolls are inserted, no shift of the mandible is observed.

In this study, these subjects without TMD's symptoms were investigated not in usual conditions, but after an experimentally induced imbalance of occlusion, given by a unilateral cotton roll placed in the right or the left sides. The condition "in habitual occlusion" was used as control.

### The variables

The platform was the Electronic Baropodometer MultiSensor system, D.B.I.S. (Digital Biometry Images Scanning) (Diagnostic Support S.r.l. Via Dora 1 - 00198 Roma, Italy) (Figure 1).

The position of the load cells could not be modified to adapt to different foot shapes. However, the differences in foot shapes among the subjects were never indicated as a limit for the study of the loading in the orthopaedic literature, and it is why we adopted this method in this investigation [9].

The variables investigated in this study were: the load surface (expressed in mm^2^), the loading pressure (expressed in gr/cm^2^) and the loading (expressed in percentage for the two sides) on the feet during locomotion in habitual occlusion and with an unilateral cotton roll interposed between dental arches in the right or in the left sides.

Posturographic recordings were performed with subjects walking on the platform and always making two walks, involving firstly the right foot and finally the left foot.

Three different conditions were enrolled during the walking posturography: i) cotton roll between the dental arches only in the left side; ii) cotton roll between the dental arches only in the right side; iii) habitual occlusion.

The experimentally induced imbalance of occlusion was performed with an unilateral cotton roll midly crushed between the dental arches in one side; in this condition the subject was asked to swallow [10]. The condition in habitual occlusion was used as control condition.

For each condition, the mean pressure during walking (gr/cm^2^) on the theoretical barycentre, the percentage of loading on the left and the right feet and the loading surface (mm^2^) on the right and the left feet, were recorded as posturographic parameters. The theoretical barycentre was considered as the point whose projection on the ground falls in the middle of the connecting segment between the barycentre of the right and left limbs [10].

### Data analysis

The Statistical Package for Social Sciences programme (SPSS^® ^Inc., Chicago, Illinois, USA) was used to perform the data analysis. Each data set was tested for normality using the Skewness test and the Kurtosis test. Through these analyses, parametric methods were used for the hypothesis testing. For each variable, a one way ANOVA was performed to evaluate the existence of significant differences among the three different experimental conditions. Subsequently, and when appropriate, pairwise comparisons, between the experimental conditions were performed by a Tukey HSD and a Bonferroni test. A p value less than 0.05 was accepted for rejection of the null hypothesis.

## Results

The pressure on the theoretical barycentre showed no statistically significant difference among the experimental conditions (Table 1). When a single cotton roll was positioned in the right dental arch, the percentage of loading (Table 2) on the right foot resulted significantly lower than in habitual occlusion or with cotton roll in the other side (p < 0.001).

**Table 1:** Pressure (gr/cm^2^) in the various experimented conditions

	Experimentally induced imbalance of occlusion given by a cotton roll in the left side	Experimentally induced imbalance of occlusion given by a cotton roll in the right side	Habitual occlusion
Mean	1280.0	1340.0	1320.0
Standard deviation	365.05	354.32	363.27
Maximum	2000	1920	2100
Minimum	688	650	630

**Table 2:** Right loading (%) in the three experimented conditions

	Experimentally induced imbalance of occlusion given by a cotton roll in the left side	Experimentally induced imbalance of occlusion given by a cotton roll in the right side	Habitual occlusion
Mean	55.8	36.2 *	51.7
Standard deviation	8.06	4.40	5.68
Maximum	89	44	64
Minimum	36	29	37

* Significantly lower than the other conditions

In addition, when a single cotton roll was positioned in the left dental arch, the percentage of loading (Table 3) and the loading surface (Table 4) on the left foot resulted significantly lower than in the other conditions (p < 0.001).

**Table 3:** Left loading (%) in the three experimented conditions

	Experimentally induced imbalance of occlusion given by a cotton roll in the left side	Experimentally induced imbalance of occlusion given by a cotton roll in the right side	Habitual occlusion
Mean	26.9 *	52.6	51.7
Standard deviation	5.03	5.08	5.07
Maximum	46	65	65
Minimum	15	42	36

* Significantly lower than the other conditions

**Table 4:** Left surface (mm^2^) in the three experimented conditions

	Experimentally induced imbalance of occlusion given by a cotton roll in the left side	Experimentally induced imbalance of occlusion given by a cotton roll in the right side	Habitual occlusion
Mean	43.1 *	72.5	75.2
Standard deviation	5.2	9.45	9.2
Maximum	65	101	109
Minimum	33	53	58

* Significantly lower than the other conditions

Finally, when a single cotton roll was positioned in the right dental arch, the loading surface (Table 5) on the right foot resulted significantly lower than in habitual occlusion or with cotton roll in the other side (p < 0.001).

**Table 5:** Right surface (mm^2^) in the three experimented conditions

	Experimentally induced imbalance of occlusion given by a cotton roll in the left side	Experimentally induced imbalance of occlusion given by a cotton roll in the right side	Habitual occlusion
Mean	80.2	42.8 *	76.5
Standard deviation	9.6	3.7	10.9
Maximum	98	51	106
Minimum	60	40	58

* Significantly lower than the other conditions

## Discussion

In this study, a few parameters were used to properly describe posture during walking, according to the concept that the locomotion of Humans, is described by different parameters [11]. Each of the considered parameters represents a different aspect of posture.

The mean pressure describes the entity of load that is on the theoretical barycentre of the patient, while the percentage of load and loading area surface, for each foot, clarifies the entity and the existence of an imbalance between the right and the left sides during locomotion.

In this study, in a sample of health subjects without TMD's symptoms, an experimentally induced imbalance (a cotton roll interposed between dental arches in the right or the left sides) resulted associated to a change in the loads on feet during walking (Tables 2, 3, 4 and 5).

In this study, only females were included in the sample; this was done because of the different postural attitude and walking attitude observed between genders in several studies. On comparing the genders, males and females [12] showed a significantly different walking speed and cadence. In that study, after excluding the effect of the walking speed and body size, the most prominent gender difference in the kinematic data of trunk motion is believed to be the more extended trunk posture in women. Another previous study [13] suggested that the female pelvis is more anteriorly tilted throughout the gait cycle, also suggesting a different attitude during walking between genders.

These studies might explain the inclusion of only females in our investigation. The differences in postural loadings between genders will be studies through further investigation, but this topic is beyond the scope of this study.

In this study, the cotton rolls were inserted between the dental arches not to distribute the occlusal load over several teeth and minimize the impact of incongruous dental contacts, [14] but to create an immediate slow imbalance of occlusion when the cotton roll was inserted in one of the two sides, that was associated also to a change in the activity of masticatory muscles,[15] and neck muscles [16].

In a previous study [1], we found that subjects with TMD's symptoms showed some differences in the distribution of loads on feet, when two cotton rolls were interposed between the dental arches, respect to health subjects. These previous findings suggested some interrelationships between occlusion and locomotion in subjects with TMD's symptoms, but failed to clarify whether these relationships are absent in subjects without TMD's symptoms. Thus, through this new protocol, we found the presence of correlations between occlusion and locomotion also in health subjects without TMD's symptoms; specifically, we observed in health subjects without TMD's symptoms that an experimentally induced imbalance of occlusion can generate an imbalance of load distribution on feet during walking.

In this study, no condition was tested with deprivation of any interference from visual sensory functionand this could have limited the evaluation of the postural control; this limitation of the study was due to the fact that during walking, the visual sensory function tends to be necessary for the explication of movements [17]. Thus, this elimination could have probably complicated the interpretations of the observed findings.

## Conclusion

In an adult caucasian sample of females without TMD's symptoms, the percentage of loading and the loading surface on the right and the left feet resulted to be influenced by an experimentally induced imbalance of occlusion given by a single cotton roll positioned in the right or the left dental arches, respectively. Specifically, when a single cotton roll was positioned in the right dental arch, the percentage of loading and the loading surface on the right foot resulted significantly lower than in habitual occlusion.

In addition, when a single cotton roll was positioned in the left dental arch, the percentage of loading and the loading surface on the left foot resulted significantly lower than in habitual occlusion (p < 0.001).

The present findings provided some insights about the association of stomatognathic inputs and locomotion, evidencing that also in health subjects without TMD's symptoms, there are detectable interrelationships between occlusion and locomotion, that were not found in a previous study from the same researchers [1].

## Authors' information

Antonella Polimeni is a Full Professor at University La Sapienza and Matteo Saccucci is a Staff member at the Unit of Orthodontics at University La Sapienza.

## Competing interests

The authors declare that they have no competing interests.

## Authors' contributions

ST (first author) conceived the study, carried out the analysis of posturographic data, participated in the selection of subjects and performed the statistical analysis; she drafted the manuscript. FF revised the final manuscript. All authors read and approved the final manuscript.
